# The Effect of Dry Eye Disease Treatment Prior to Cataract Surgery on Refractive Error Reduction

**DOI:** 10.3390/jcm15041640

**Published:** 2026-02-21

**Authors:** Katarzyna Biela, Mateusz Winiarczyk, Beata Gumieniak-Goch, Jerzy Mackiewicz

**Affiliations:** 1 Department of Vitreoretinal Surgery, Medical University of Lublin, Chmielna 1, 20079 Lublin, Poland; 2 Department of Ophthalmology, Provincial Hospital in Zamosc, al. John Paul II 10, 22400 Zamosc, Poland

**Keywords:** dry eye disease, moisturizing drops, refractive outcome, intraocular lens power calculation, cataract surgery

## Abstract

**Background/Objectives**: Dry-eye disease (DED) is a disorder of the eye surface associated, among other things, with tear film instability. It can lead to abnormal biometry results, especially with respect to keratometry. DED is more common in the elderly population. Its prevalence is often underestimated. Failure to provide adequate treatment prior to biometry may result in refractive errors after cataract surgery. The purpose of this study was to quantify the impact of DED on refractive predictability in cataract surgery and assess whether short, preoperative ocular-surface optimization reduces the mean absolute error (MAE) of postoperative refraction, regardless of DED. **Methods**: Seventy patients undergoing cataract surgery were divided into three groups: A—individuals with DED who were receiving treatment; B—individuals without DED who were receiving treatment; and C—a control group. In all groups, biometry was performed twice, before and after treatment (groups A and B) or at two-week intervals without treatment (group C). All of the individuals underwent cataract surgery. Refractive error was calculated one month after surgery for both biometry measurements (before and after treatment). **Results**: After dry eye treatment, a reduction in refractive error was achieved in both groups with and without DED. The MAE in the group with DED was 0.39 ± 0.31 vs. 0.27 ± 0.30 (*p* < 0.001), and the MAE for those without DED was 0.30 ± 0.25 vs. 0.24 ± 0.20 (*p* = 0.043). No significant differences in biometric measurements were observed in any of the groups. The most variable parameter was corneal astigmatism in the DED group. **Conclusions**: Proper preparation of the eye surface for biometric measurement reduces refractive errors after surgery.

## 1. Introduction

According to WHO reports, cataracts are the most common cause of preventable blindness (15.2 million) and the second most common cause of moderate and severe vision impairment (MSVI), with 78.8 million people in the patient population being over 50 years of age [1]. Nowadays, cataract surgery is the most commonly performed ophthalmic surgery, and the number of procedures performed reaches about 5 million per year in Europe [2]. Globally, the number approaches 20 million a year [3].

Objective assessment of the effectiveness of the procedure is based on best corrected visual acuity (BCVA) and refraction prediction error (RPE), otherwise known as mean error (ME) [4]. ME is the difference between the predicted and actual spherical equivalent of postoperative refraction [5]. The actual postoperative refractive equivalent is the correction value at which the patient achieves BCVA [6]. An assessment is usually performed one month after surgery [7]. The predicted refractive equivalent is the value calculated from biometry. An ME above 1 D is considered a significant refractive error [8].

As mentioned above, biometry is required to calculate the predicted refractive equivalent. This test allows the power of the intraocular lens (IOL), implanted during cataract surgery, to be calculated. The gold standard for biometry is non-contact optical biometry [9]. In this study, we use keratometry, axial length, and, in some formulas, anterior chamber depth to calculate IOL power. An accurate keratometry measurement in optical biometry depends strongly on the quality of the tear film. The measurement is based on the reflection of light precisely from the surface of the tear film, whose poor quality can interfere with measurements [10]. A 1 D error in preoperative biometry corresponds to a 1 D refractive error postoperatively [11]. One of the more common causes of errors in keratometry is dry eye disease (DED).

According to TFOS DEWS III, dry eye is a multifactorial, symptomatic disease characterized by a loss of homeostasis of the tear film and/or ocular surface, in which tear film instability and hyperosmolarity, ocular surface inflammation and damage, and neurosensory abnormalities are etiological factors [12]. Tear film instability, a component of DED, results in abnormal keratometry measurements [13].

The importance of correct diagnosis and treatment of DED prior to surgery is particularly emphasized in the context of refractive surgery [14,15]. However, the problem of dry eye is often underestimated in regard to patients with cataracts [16]. This leads to a lack of adequate treatment prior to biometry, which is critical for a satisfactory refractive outcome. It is important to note that DED is more common in the elderly patient population, and the same age group is also more likely to be affected by cataracts [1,17]. It is worth emphasizing that DED may affect the refractive effect of cataract surgery—higher RPE values and a reduction in these values after treatment have been observed for patients with DED [18,19,20,21,22].

The aim of the study was to quantify the impact of DED on refractive predictability in cataract surgery and assess whether short, preoperative ocular-surface optimization reduces the mean absolute error (MAE) of postoperative refraction, regardless of the presence of DED.

## 2. Materials and Methods

**Figure 1:**
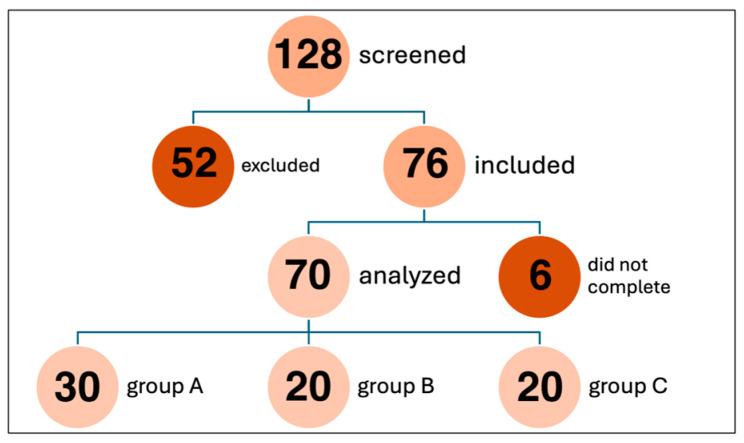
Selection process for the study.

The study included 128 participants undergoing cataract surgery in 2022–2024. Fifty-two patients met the exclusion criteria, such as an ocular axial length < 21.5 mm or >26 mm, a previously diagnosed ocular surface disorder, the use of any eye drops in the two weeks prior to the study, and the need to use other drops in the preoperative period. The aforementioned factors may affect the outcome of postoperative refraction. Seventy-six patients were included in the study. Of these, six patients did not complete the study due to cancellation of surgery or a change in IOL choice. The study ultimately included seventy participants undergoing cataract surgery, consisting of thirty-three women and thirty-seven men. The mean age of the subjects was 72.93 ± 6.88 years. The selection process for the study is presented in Figure 1.

During the qualifying visit for cataract surgery, following acquisition of informed consent, a preliminary examination was performed. Visual acuity was assessed based on Snellen tables, and screening for DED was performed according to TFOS DEWS II criteria [23]. The criteria for diagnosing DED are presented in Table 1.

Subjective symptom assessment was performed using the ocular surface disease index (OSDI) form. Tear film osmolarity was tested with an I-PEN device (I-Med Pharma, Canada). Corneal staining with fluorescein and evaluation of tear film break-up time (TBUT) were performed. Based on the outcome, patients were divided into 3 groups. Group one (A, n = 30) consisted of patients with a preoperative diagnosis of DED. To evaluate the refractive effect of eye surface preparation regardless of DED, patients who did not meet the diagnosis criteria were divided into two subgroups without randomization (group B, n = 20; group C, n = 20). Inclusion in group B or C was determined by the order in which patients registered for the examination (patients were assigned alternately).

Treatment of DED, performed according to the TFOS DEWS II and Polish Ophthalmological Society guidelines, was applied in group A (patients with DED) and group B (patients without DED). Treatment included the use of trehalose moisturizing drops 4 times a day and an eyelid margin hygiene twice a day. Treatment was administered for 2 weeks prior to admission for cataract surgery. In group C (the control, without DED), no interventions were employed before surgery.

All patients were subjected to biometry twice with an IOL Master500 (Zeiss, Oberkochen, Germany). The first measurement was taken during qualification, prior to testing for DED. The second measurement was taken on the day of surgery, after 2 weeks of treatment (group A and B) or follow-up (group C). IOL power was calculated based on the Haigis formula. Cataract surgery was performed by a single operator. A hydrophobic spherical monofocal intraocular lens (Hoya iSert^®^ 150; Hoya Surgical Optics GmbH, Frankfurt/Main, Germany) was implanted in all patients. Four weeks after surgery, visual acuity and postoperative refraction were assessed, and the ME was calculated.

The primary endpoint was defined as a change in MAE. Secondary endpoints included differences in DED diagnostic tests; changes in keratometry after treatment; differences in MAE intervals of 0.25 D, 0.5 D, and 0.75 D; and changes in visual acuity after surgery.

The results obtained were subjected to statistical analysis (Statistica 13.3, StatSoft, Poland). The values of the quantitative variables analyzed are presented as means, medians, lower and upper quartiles, minimum and maximum values, and standard deviations, and the qualitative variables are presented as counts and percentages. The Chi^2^ test was used to assess the correlation of qualitative variables. The normality of the distribution of the variables in the study groups was checked using the Shapiro–Wilk normality test. An analysis of variance was used to examine the differences between the groups. Differences between measurements from the qualifying visit and measurements on the day of surgery were assessed using the Student’s *t*-test for dependent samples and, if the conditions for its application were not met, the Wilcoxon paired rank-sum test. A significance level of *p* < 0.05 was assumed.

## 3. Results

**Figure 2:**
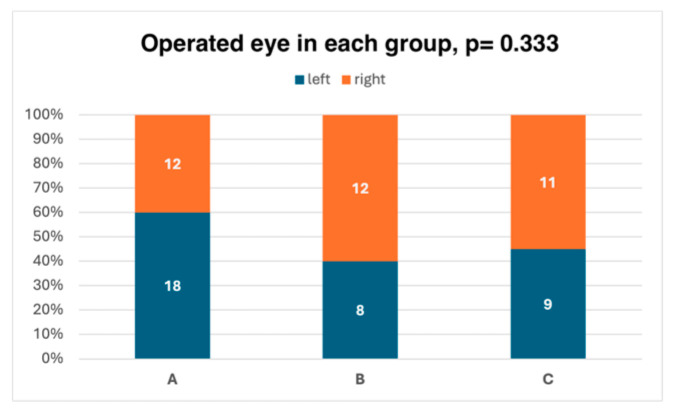
Eye operated on in each group. The groups did not differ in terms of the eye operated on. Significance level for the analysis-of-variance test: *p* < 0.05.

**Figure 3:**
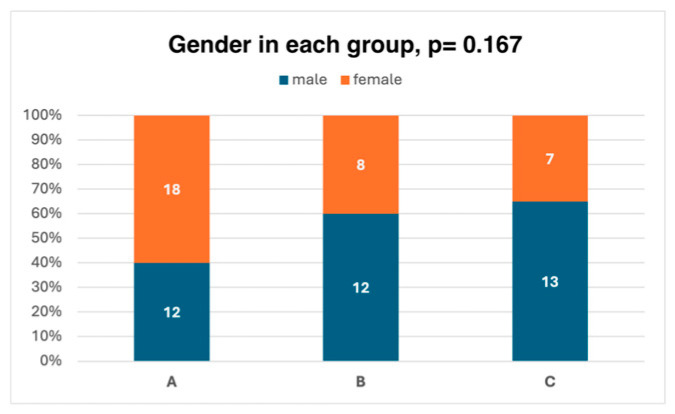
Gender in each group. The groups did not differ in terms of gender. Significance level for the analysis-of-variance test: *p* < 0.05.

There were no statistically significant differences between the groups in terms of age, gender, the eye operated on, or visual acuity both before and after surgery. The demographic characteristics of the groups are presented in Table 2 and in Figure 2 and Figure 3.

Based on the diagnostic criteria, DED was diagnosed in 30/70 patients (42.86%). Compared with the non-DED groups, the DED group had higher OSDI scores, lower TBUT values, and more corneal staining; tear osmolarity did not differ significantly across groups.

In group A, OSDI scores were significantly higher (21 ± 6 in group A, 6.25 ± 2.82 in group B, and 6.0 ± 3.88 in group C (*p* < 0.001)). Patients in this group also had a significantly higher score of surface staining of the eye operated on based on the Oxford scale (1.1 ± 0.8 in group A, 0.5 ± 0.76 in group B, and 0.35 ± 0.50 in group C (*p* < 0.001)). TBUT was significantly lower (8.00 ± 2.16 in group A, 10.15 ± 1.60 in group B, and 10.1 ± 1.77 in group C (*p* = 0.007)). The patient groups without a diagnosis of DED (group B and C) showed no significant differences in OSDI, TBUT, or staining.

Surprisingly, tear film osmolarity did not differ significantly between groups (297.6 ± 19.19 in group A, 296.7 ± 15.02 in group B, and 292.05 ± 10.73 in group C; *p* = 0.74).

The results of the diagnostic tests for each group are shown in Table 3.

For all patients, cataract surgery proceeded without complications. There was an improvement in best corrected visual acuity (BCVA) after surgery in all groups. In group A, BCVA before surgery was 0.35 ± 0.15; after surgery, it was 0.88 ± 0.24, *p* < 0.001. In group B, BCVA before surgery was 0.40 ± 0.13; after surgery, it was 0.96 ± 0.11 (*p* < 0.001). In group C, BCVA before surgery was 0.43 ± 0.17, while that after surgery was 0.95 ± 0.08 (*p* < 0.001).

There were no significant differences in any of the biometric measurements (keratometry, axial length, and anterior chamber depth) in any of the groups. It is noteworthy that the most variable parameter in group A, the group with DED, was corneal astigmatism (the value before ocular-surface optimization was 0.81 D ± 0.46 D, while that afterwards was 0.91 D ± 0.57 D; *p* = 0.076). In the other groups, the results were not statistically significant (in group B, 0.84 ± 0.56 vs. 0.86 ± 0.48 (*p* = 0.727); in group C, 0.76 ± 0.42 vs. 0.82 ± 0.32 (*p* = 0.208)).

After determining the magnitude of postoperative refraction, the MAE was calculated for measurements taken at the qualifying visit (MAE I) and immediately before surgery (MAE II). Group A exhibited a significant reduction in MAE after DED treatment (0.39 ± 0.31 vs. 0.27 ± 0.30, p<0.001). Interestingly, MAE also significantly decreased in group B (0.30 ± 0.25 vs. 0.24 ± 0.20; p= 0.043). No significance was reached in group C (0.27 ± 0.17 vs. 0.28 ± 0.19; p= 0.539).

All groups achieved a satisfactory MAE percentage in the range of ±0.25 D and ±0.50 D. In group A, 90% of patients achieved an MAE in the range of ±0.75 D, and in group B, 95% achieved such an MAE, while in group C, this result was achieved for all patients (100%). The results are presented in Table 4.

## 4. Discussion

### 4.1. No Effect of Osmolarity on Results

Numerous reports have highlighted the relationship between increased tear osmolarity and keratometry measurement errors. Epitropoulus et al. found greater variability in K in patients with tear hyperosmolarity [24]. The mean difference in the average K was 0.13 D in the normal group and 0.28 D in the hyperosmolar group. This resulted in a statistically significant difference in IOL power > 0.5 D in the hyperosmolar group compared to normal tear osmolarity [24]. Increased tear osmolarity is listed as one of the criteria for the diagnosis of DED [12]. It is also used in screening tests to diagnose DED before refractive surgery [25]. A surprising finding in this study was the lack of statistical significance of tear osmolarity measurements between the groups. In our study, we used an I-Pen osmometer to measure tear osmolarity. Despite FDA approval, there have been differences between assessments of the clinical usefulness of this device. The I-Pen showed good sensitivity and specificity in both in vitro and in vivo studies [26,27,28]. Higher tear osmolarity is not a parameter specific to DED alone—higher values were also observed in people with increased BMI and in smokers [29,30]. Shimazaki et al. obtained results consistent with our studies, where the I-Pen was not associated with higher tear osmolarity values in the DED group compared to the healthy group [31].

Tear osmolarity measurement is an important factor in the diagnosis of DED, and it is one of the criteria for diagnosing the disease in the TFOS DEWS III guidelines [12]. According to research by Wolffsohn et al., the sensitivity of DED diagnosis in this model is 87.7%. Omitting tear osmolarity from the diagnostic criteria results in a sensitivity reduction of <5%. [32]. Asian societies, on the other hand, recommend considering only symptoms and TBUT as an easily accessible marker of tear film instability [33,34,35]. In the study group, 75% of asymptomatic patients (OSDI < 13) had a positive result in at least one diagnostic test for DED (14/20 in group B, 15/20 in group C). It therefore appears that the lack of significance in the tear osmolarity test did not greatly affect the detectability of the disease in this study. However, tear osmolarity should not be the only parameter assessed in the diagnosis of DED.

### 4.2. Changes in Corneal Astigmatism in Patients with DED

In this study, we did not find any significant differences in biometric measurements between the individual groups. However, it is worth noting that the greatest differences, at the threshold of statistical significance, were observed in corneal astigmatism in the group of patients with DED (0.81 ± 0.46 vs. 0.91 ± 0.57, with *p* = 0.076). As mentioned earlier, accurate keratometry depends on the quality of the tear film, which reflects light during measurement [36]. The impact of DED on keratometry results has been proven. Ahn et al. found significantly higher variability in corneal astigmatism in patients with DED. Patients with a variability >0.25 D also had a significantly higher risk of refractive surprise > 0.5 D—45.5% vs. 17.9% [37]. Yang et al. found greater variability in mean K difference in a DED patient group. In this group, variability >0.5 D was also observed more frequently. Interestingly, these results did not correlate with NIBUT values or OSD SPEED II test results [38]. The same authors evaluated other biometric parameters in another study and found no differences between them [39]. Our patients received monofocal IOL implants, but it should be remembered that similar changes in astigmatism in the DED group may be observed in patients who have undergone planned toric lens implantation.

### 4.3. Changes in MAE

In our study, we observed a reduction in MAE after DED treatment. There are several reports confirming refractive benefits after treatment in the period leading up to cataract surgery. Kim et al. used 0.5% loteprednol eye drops, 0.05% cyclosporine A eye drops, and eyelid margin hygiene in pre-biometry treatment. They compared the results with findings derived from a control group that received only artificial tears. The study group achieved an MAE of 0.24 ± 0.19 D, while the control group achieved 0.38 ± 0.34 D, and the difference was statistically significant [21]. Hovanesian et al. evaluated the effect of using 0.09% cyclosporine A eye drops prior to biometry. The study group consisted of patients with DED whose eyes were measured twice—before and after treatment. They achieved a statistically significant reduction in MAE from 0.39 ± 0. 30 D to 0.33 ± 0.25 D [20]. In a second study, the same authors evaluated the efficacy of 5% lifitegrast for this indication. However, they did not evaluate the change in MAE; they only evaluated the percentage of patients with MAE in the range of ±0.25 D, ±0.5 D, and ±0.75 D for measurements before and after treatment. In these ranges, 47%, 71%, and 81% of patients were obtained before treatment and 50%, 79%, and 91% were obtained after treatment, respectively, and all differences were statistically significant [19].

Nilsen et al. used artificial tears in a DED group for two weeks prior to biometry and compared the results with an untreated DED group and a control group consisting of patients without DED. In this study, no significant differences were found between the groups in terms of the predictability of postoperative refraction [40]. It seems that eyelid margin hygiene is also required to protect the surface of the eye and obtain stable biometric measurements. This is confirmed by individual reports assessing the effect of the Thermal Pulsation System on changes in astigmatism power and axis. The variability of astigmatism in these studies was 64–68% [41,42]. Therefore, a comprehensive approach seems necessary, covering both excessive evaporation and insufficient tear production in DED.

A comparison with results obtained by other authors is presented in Table 5.

It is worth noting that the recommendations do not specify a minimum refractive error. Norrby estimated the minimum MAE to be between 0.36 and 0.4 D [6]. Nowadays, lower values can be achieved [43,44,45,46]. The parameter most commonly used to assess the refractive effectiveness of the procedure is the percentage of patients achieving MAE within the range of ±0.5 D and ±1 D, assuming a good refractive result for 60% of patients ±0.5 D and 90% ± 1 D [9,47]. The limits of refractive effectiveness are constantly shifting. Currently, satisfactory refractive results are achieved through a combination of small changes in the surgical protocol, such as the use of newer equipment, changes in the calculation formula, and appropriate patient preparation [44,48,49].

### 4.4. How to Optimize the Procedure (with Reference to Current Guidelines)?

Based on current research and recommendations, three procedures can be considered prior to cataract surgery: full DED diagnosis for each patient, application of eye drops immediately prior to biometry, and preparation of the eye surface for all patients.

The need to exclude or treat DED prior to refractive surgery has been discussed for a long time. The ASCRS recommends DED diagnosis for all patients prior to such procedures. Screening includes an interview based on the OSD SPEED II form and two bedside tests: tear osmolarity and metalloproteinase 9. If significant disorders are found, the procedure should be postponed and appropriate treatment implemented [25]. The AAO mentions the need to assess the eyelids and the anterior surface of the eye, especially in older patients undergoing cataract surgery [50]. Such a procedure seems to reduce not only the risk of refractive error but also the severity of DED after surgery [51,52,53]. In contrast to these guidelines, Gauthier et al. observed a higher incidence of SE errors or a cylinder > 1.0 D in patients after toric lens implantation only in cases of keratoconus or high astigmatism or after LASIK or radial keratotomy. DED was not relevant. It is worth noting that DED was diagnosed in this study only on the basis of corneal staining [54]. The question of whether an error of 1.0 D can be considered sufficient for the patient and the surgeon also remains.

Some studies have suggested applying moisturizing eye drops immediately before biometric measurement. This is due to the greater variability of keratometry measurements among patients with DED, as mentioned earlier [13,38]. Rochet et al. assessed the impact of this procedure on keratometry variability and refractive error in patients with toric IOL implants. Overall, 43.8% required a change in IOL power, and 17.7% required a change in the IOL axis of more than 10 degrees. The changes were significant in patients with TBUT < 5 s. A significant reduction in MAE was also achieved in these patients—from 0.48 ± 0.50 D to 0.37 ± 0.25 D [55]. In contrast, Jensen et al. did not obtain significant variability in biometry measurements performed without and after the application of eye drops [56]. However, there is no information on whether these were patients with or without DED. It is worth mentioning the study by Röggla et al., in which a series of biometric measurements were performed after applying moisturizing eye drops. After 30 s, a change in keratometry of more than 0.5 D was observed in 13.2% of normal eyes and 27.8–34% (depending on the drops) in eyes with DED [57]. Although the authors of the study recommend measurement without drops, it is worth noting that there was no MAE assessment. Therefore, it is not possible to determine which results gave a smaller error.

One procedure that may be considered is the use of moisturizing drops and eyelid margin hygiene in all subjects prior to biometry, regardless of DED diagnosis. In this study, this procedure reduced MAE from 0.30 ± 0.25 D to 0.23 ± 0.20 D (*p* = 0.043). It is worth noting that statistics on people with DED undergoing cataract surgery may be underestimated. In the PHACO study, which assessed this issue, 54% of participants had a positive OSDI questionnaire result, and in the asymptomatic group, 85% had an abnormal osmolarity or MMP-9 test result, suggesting DED. Overall, 80% had at least one result suggesting DED [16]. In this study, DED was diagnosed in 42.86%. At least one result suggesting DED was found in 85.71% of patients, while in the asymptomatic group, 75% of patients had at least one positive diagnostic test result. The results are therefore consistent with previous reports, and a comparison is presented in Table 6.

### 4.5. Limitations of the Study

This study is not without limitations. There was no randomization in the assignment of subjects to groups B and C. In groups A and B, compliance was declarative, without re-evaluation of DED signs. We used IOL Master 500 and the Haigis formula in our calculations. Swept source devices demonstrate higher biometric measurement accuracy, but no differences were observed in predicting postoperative refraction [58,59]. However, significant fluctuations were observed in keratometry measurements, especially when using the SRK-T formula [60,61]. Our research confirms that stabilizing the surface of the eye improves the predictability of refractive results, and this effect should be maintained regardless of the formula used.

## 5. Conclusions

DED is a common condition in patients undergoing cataract surgery. Proper preparation of the surface of the eye for biometric measurement can help reduce refractive errors after surgery. Further research is needed to optimize preoperative management.

## Figures and Tables

**Table 1:** Criteria for diagnosing DED applied in the studies, based on TFOS DEWS II.

	Symptoms	Signs		
Test	OSDI	TBUT	Osmolarity	Eye Surface Staining
Abnormal result	≥13	<10 s	≥308 mOsm/L OR difference between eyes > 8 mOsm/L	>5 corneal epithelial defects OR >9 conjunctival defects OR eyelid margin epitheliopathy
Diagnosis of DED: symptoms + at least one of the signs				

Abbreviations: OSDI, ocular surface disease index; TBUT, tear break-up time.

**Table 2:** Characteristics of the groups. No significant differences were found between the groups in terms of age, visual acuity before and after surgery, MAE I, or MAE II. Analysis-of-variance-test significance level: *p* < 0.05.

Analyzed Parameter	Group	M	Min	Max	SD	Group Comparison
Age	A	73.90	56.00	93.00	7.41	*p* = 0.327
	B	73.40	61.00	86.00	6.34	
	C	71.00	53.00	82.00	6.51	
BCVA before surgery	A	0.35	0.063	0.63	0.15	*p* = 0.213
	B	0.41	0.20	0.60	0.13	
	C	0.43	0.10	0.90	0.19	
BCVA after surgery	A	0.88	0.10	1.00	0.24	*p* = 0.357
	B	0.96	0.60	1.00	0.11	
	C	0.96	0.80	1.00	0.08	

Abbreviations: M, mean; Min, minimum value; Max, maximum value; SD, standard deviation; BCVA, best corrected visual acuity.

**Table 3:** DED diagnostic test results. Patients in group A had significantly higher OSDI scores and eye surface staining. Significantly lower TBUT was found in group A. No significant differences in osmolarity were found between the groups. Significance level for the analysis-of-variance test: *p* < 0.05.

Analyzed Variable	Group A	Group B	Group C
OSDI	21.0 ± 6.0	6.25 ± 2.82	6.0 ± 3.88
	Statistical significance between groups A and B < 0.001 A and C < 0.001		
Osmolarity of the eye operated on	297.60 ± 19.19	296.70 ± 15.02	292.05 ± 10.73
	No statistical significance (*p* = 0.742)		
Osmolarity difference between eyes	8.06 ± 6.37	6.35 ± 4.03	6.15 ± 2.92
	No statistical significance (*p* = 0.677)		
TBUT of the eye operated on	8.00 ± 2.16	10.15 ± 1.60	10.10 ± 1.77
	Statistical significance between groups A and B 0.004 A and C 0.007		
Eye surface staining—Oxford scale	1.10 ± 0.80	0.50 ± 0.76	0.35 ± 0.50
	Statistical significance between groups A and B 0.026 A and C 0.006		

Abbreviations: OSDI, ocular surface disease index; TBUT, tear break-up time.

**Table 4:** MAE in the range ± 0.25 D, ± 0.50 D, and ± 0.75 D in each group. MAE I—error calculated based on first measurement. MAE II—error calculated based on second measurements (after treatment/control). Student’s *t*-test for dependent samples (MAE I vs. MAE II) and McNemar’s test (comparison of MAE intervals).

	Group A		Group B		Group C	
	MAE I	MAE II	MAE I	MAE II	MAE I	MAE II
MAE	0.39 ± 0.31 D	0.27 ± 0.30 D	0.30 ± 0.25 D	0.24 ± 0.20 D	0.27 ± 0.17 D	0.28 ± 0.19 D
	*p* < 0.001		*p* = 0.043		*p* = 0.539	
±0.25 D	43.33%	63.33%	50%	60%	50%	50%
	*p* = 0.120		*p* = 0.751		*p* = 1.000	
±0.50 D	66.67%	86.67%	85%	95%	95%	85%
	*p* = 0.127		*p* = 0.598		*p* = 0.598	
±0.75 D	86.67%	90%	95%	95%	100%	100%
	*p* = 1.000		*p* = 0.468		N/A	

**Table 5:** Comparison of MAE results with those obtained in other publications. The obtained results are consistent with previous reports.

	Hovanesian et al. CsA [20]		Kim et al. [21]		Hovanesian et al. Lifitegrast [19]		Biela et al.	
	I	II	I	II	I	II	I	II
MAE	0.39 ± 0.30 D	0.33 ± 0.25 D	0.38 ± 0.34 D	0.24 ± 0.19 D	N/A	N/A	0.39 ± 0.31 D	0.27 ± 0.30 D
	*p* < 0.03		*p* = 0.008		N/A		*p* < 0.001	
±0.25 D	41%	47%	36.6%	58.5%	47%	50%	43.33%	63.33%
	*p* < 0.05		*p* = 0.02		*p* < 0.04		*p* = 0.120	
±0.50 D	72%	73%	73.2%	90.5%	71%	79%	66.67%	86.67%
	*p* < 0.31		*p* = 0.154		*p* < 0.04		*p* = 0.127	
±0.75 D	88%	95%	85%	98%	81%	91%	86.67%	90%
	*p* < 0.03		N/A		*p* < 0.04		*p* = 1.000	

Abbreviations: MAE, mean absolute error; CsA, cyclosporine A.

**Table 6:** Comparison of DED diagnostic test results with the results of the PHACO study. The results obtained in our study are consistent with previous reports.

	PHACO Study [16]	Biela et al.
Diagnosis of DED	22% before study	42,86% in the study
Positive OSDI	54%	44.28%
At least one positive result	80%	85.71%
At least one positive result—asymptomatic group	85%	75%

## Data Availability

The data presented in this study are available on request from the corresponding author due to personal data protection.
